# Effects of graphene and CNT nanoparticle additives on combustion, performance, and emissions of a diesel engine under varying injection pressures

**DOI:** 10.1038/s41598-026-48971-9

**Published:** 2026-04-19

**Authors:** R. Sebastin Nesa Raj, B. Stanly Jones Retnam, M. Dev Anand, R. R. Neela Rajan, M. Anish, J. R. Deepak, Ajay Kumar, Ali El-Rayyes

**Affiliations:** 1 Department of Mechanical Engineering, Noorul Islam Centre for Higher Education, Thuckalay, Kanyakumari, India; 2 Department of Aeronautical Engineering, Noorul Islam Centre for Higher Education, Thuckalay, Kanyakumari, India; 3 School of Mechanical Engineering, Sathyabama Institute of Science and Technology, Chennai, India; 4 Department of Mechanical Engineering, School of Engineering, Faculty of Science, Technology and Architecture, Manipal University Jaipur, Jaipur, Rajasthan 303007 India; 5 Center for Scientific Research and Entrepreneurship, Northern Border University, Arar, 73213 Saudi Arabia; 6 Chemistry Department, College of Science, Northern Border University, Arar, Saudi Arabia

**Keywords:** Carbon nanotube, Emissions, Graphene, Hydrocarbons, Waste cooking oil, Energy science and technology, Engineering, Environmental sciences, Nanoscience and technology

## Abstract

This study investigates the influence of fuel injection pressure and nanoparticle additives on the combustion, performance, and emissions of a CI diesel engine fuelled with diesel–waste cooking oil biodiesel blends. Injection pressure was varied between 180 and 240 bar, and graphene Nano platelets (GNPs) and carbon nanotubes (CNTs) were added at 50 ppm. Increasing the injection pressure to 240 bar improved combustion quality and raised brake thermal efficiency (BTE) by 2.1%, while reducing brake-specific fuel consumption relative to lower pressures. The graphene-based blend (W20G3) exhibited the most significant emission benefits, achieving a 68% reduction in HC, 4.6% reduction in CO, and 2.5% reduction in NOx compared to baseline biodiesel at the same injection pressure. Meanwhile, the CNT-modified blend (W20C3) produced the largest improvement in smoke opacity, achieving a 44.6% reduction due to enhanced soot oxidation associated with its tubular morphology. The results confirm that graphene is more effective in reducing gaseous pollutants, whereas CNTs excel in reducing smoke, and that an injection pressure of 240 bar optimizes nanoparticle-assisted combustion. These findings provide quantitative evidence for improving biodiesel formulations and fuel injection strategies to achieve cleaner and more efficient diesel engine operation.

## Introduction

Fossil-derived energy sources such as diesel, compressed natural gas (CNG), gasoline, and liquefied petroleum gas (LPG) continue to dominate the transportation sector, collectively contributing to nearly 99.9% of its overall fuel utilization^1^. While these conventional fuels have supported global mobility and economic growth, there is growing concern about the long-term sustainability of this reliance. A major issue is the continuous depletion of fossil fuel reserves, which threatens energy security and price stability^2–4^. Beyond supply challenges, the environmental impacts of fossil fuel combustion are increasingly alarming. Most vehicles rely on internal combustion engines that emit significant quantities of harmful pollutants such as CO₂, CO, NOₓ, HC, and PM. For example, the oxidation of 1 L of diesel results in the emission of approximately 2.9 kg of carbon dioxide equivalents (CO₂) into the atmosphere, which substantially contribute to climate change and global warming^1^.

Data from 2018 indicate that an estimated 20% of global energy resources are utilized by the transportation sector^5^, producing approximately 23% of worldwide carbon dioxide emissions and 14% of overall greenhouse gas emissions. Given this significant environmental footprint, there is an urgent need to transition toward cleaner, more sustainable fuel alternatives^6^. Overdependence on fossil fuels also has serious health and ecological consequences. Traffic emissions substantially contribute to the decline in air quality within densely populated areas, linked to respiratory and cardiovascular diseases and a reduction in average life expectancy. Simultaneously, the accumulation of greenhouse gases disrupts ecosystems, accelerates climate change, and threatens biodiversity. Addressing these challenges requires a global shift to sustainable energy sources. Alternative fuels such as biodiesel, ethanol, hydrogen, and electricity^7^, alongside cleaner combustion technologies, provide effective strategies to reduce the environmental and public health risks linked to traditional fossil fuel consumption in the transportation sector.

Conventional diesel and biofuels, including methyl esters and alcohols, differ notably in their oxygen content—biofuels typically contain over 10% oxygen by weight, while conventional diesel has much lower oxygen levels^8–11^. An increased oxygen concentration in biofuels contributes to more thorough combustion and enhances overall engine performance. Recently, producing biofuels from waste oils has attracted attention due to its dual benefits of effective waste management and fossil fuel conservation^12–14^. Concurrently, enhancing conventional diesel with selected additives has shown significant potential in improving its performance characteristics, especially when used with diesel-biofuel blends, leading to improvements in both engine efficiency and environmental impact. Among these additives, nanoparticles have shown significant promise due to their unique physicochemical characteristics. Advances in nanotechnology have enabled large-scale production of nanoparticles, expanding their potential applications. Nanoparticles are defined as particles measuring between 1 and 100 nanometers, typically dispersed in a base fluid to form nanofluids^15^. When incorporated into pure diesel or diesel-biofuel blends, the resulting mixtures are often termed “nano diesel.” This emerging area has attracted extensive scientific and technical research. Many studies support the usage of metal oxide nanoparticles in fuel blends because of their ability to improve combustion performance and minimize the emission of hazardous exhaust pollutants^16–18^.

Carbon nanotubes (CNTs), a class of nanostructured carbon-based materials, have recently gained considerable attention for their exceptional physical, chemical, and mechanical properties. These versatile materials are widely used in advanced composites, electronics, biomedical engineering, and increasingly in energy and fuel technologies^19^. Within internal combustion engine and alternative fuel research, CNTs are investigated as highly effective nano-additives. Their inclusion in fuel formulations has demonstrated potential benefits such as enhanced combustion characteristics, increased cetane number, anti-knock effects, and cleaner, more complete combustion^20^.

Several investigations have analyzed the influence of carbon nanotubes (CNTs) on engine performance parameters, combustion efficiency, and emission mitigation. For instance, Hosseini et al.^21^ investigated the incorporation of CNTs at concentrations of 30, 60, and 90 ppm into B5 and B10 biodiesel blend formulations. Their findings showed that CNTs improved engine achieved higher engine efficiency alongside a significant decline in CO emissions, nanoscale carbon particles, and soot. However, they also observed a rise in (NOₓ) emissions with increasing CNT concentration, likely due to elevated combustion temperatures. The large surface area and excellent thermal conductivity of CNTs were identified as key factors enhancing the combustion process by accelerating enhancing reaction rates, decreasing the time to ignition, and improving the interaction between fuel and air. El-Seesy et al.^22^ determined that incorporating approximately 40 ppm of multi-walled carbon nanotubes (MWCNTs) into a B20 biodiesel blend yields notable enhancements in engine performance and emission characteristics. Similarly, Banapurmath et al.^23^ compared the Impacts of graphene, silver, and MWCNT nanoparticles at 25 and 50 ppm in biodiesel blends. Results demonstrated that the elevated surface-to-volume ratio characteristic of nanoparticles contributed significantly to improved fuel–air interaction and superior evaporation properties contributed to significant reductions in NOₓ, CO, and unburned hydrocarbons (UHC). These enhancements were primarily attributed to improved heat transfer and more effective combustion processes occurring within the engine cylinders. Ghafoori et al.^24^ further tested B20 biodiesel blends containing 2.5 to 30 ppm CNTs in diesel engine applications, enhancements of up to 17% in power output and 18% in torque were observed, along with a substantial 38.5% reduction in BSFC. Moreover, the release of unburned hydrocarbons and carbon monoxide was reduced by 22% and 18%, respectively, as a result of enhanced combustion efficiency enabled by the high thermal conductivity and catalytic activity of carbon nanotubes (CNTs).

Graphene-based nanoparticles are also noted for their high thermal conductivity and effective lubrication performance, which enhances fuel atomization and reduces engine friction, ultimately improving performance and lowering emissions^25^. Research has also shown that combining metal oxide nanoparticles with carbon-based materials such as Graphene can facilitate more efficient oxidation processes, thereby markedly decreasing emissions of UHC, CO, and NOₓ pollutants^26,27^. This study explores the role of Graphene oxide nanoparticles as diesel fuel additives, focusing on their potential to improve engine efficiency while minimizing environmental impact. Kumar et al.^28^ carried out a comprehensive study using cerium oxide nanoparticles at 80 ppm to assess the effect of varying injection pressures—180, 210, and 240 bar—on compression ignition engine efficiency. Their results demonstrated that a reduction in ignition delay significantly increases in injection pressure was found to accelerate the combustion rate of the fuel–air mixture, consequently, the overall combustion efficiency was enhanced. Moreover, elevated fuel injection pressures intensified the heat release rate (HRR) inside the combustion chamber, leading to improved engine output and reduced specific fuel consumption. The addition of metallic nanoparticles—most notably cerium oxide facilitated more thorough combustion, significantly contributing to the mitigation of toxic exhaust emissions. The use of nanoparticles as additives has gained significant recognition due to their excellent catalytic properties, smaller size and larger surface area, which improve combustion and reduce emissions.

Similarly, Lalvani et al.^29^ Explored the effect of in-cylinder turbulence on the thermal efficiency of a diesel engine. They found that increased fuel viscosity could hinder air-fuel mixing, which is critical for effective combustion. The study concluded that fine-tuning both injection pressure and turbulence intensity within the combustion chamber effectively reduces NOₓ emissions, a major environmental pollutant. Furthermore, higher injection pressures were shown to result in decreased concentrations of HC, CO, and visible smoke particles all of which negatively impact air quality. In related research, Yesilyurt et al.^30^ examined injection pressures ranging from 170 to 220 bar across different engine speeds. They reported that higher injection pressures markedly improvedBTE and engine torque, with the optimum at 210 bar, which achieved the highest BTE and lowest pollutant emissions. However, the study also found that increased cylinder pressure extended ignition delay, resulting in higher fuel consumption, highlighting the trade-offs involved in adjusting injection parameters. Nevertheless, optimizing injection pressure enhanced fuel atomization and mixing within the combustion chamber, these conditions promoted more complete and efficient fuel combustion. Additionally, it was observed that EGT exhibited an upward trend with the escalation of injection pressure, while NOₓ and CO emissions declined, likely a result of improved ignition timing and enhanced combustion efficiency and combustion stability achieved through this optimization. The study of Rao et al.^31^ shows that an increase in the injection pressure of a diesel fuel produces smaller droplet sizes which allows for a better interaction between the turbulence generated during the injection process and the combustion process; as such, the BTE is increased while decreasing emissions related to combustion at the same time. As a result of this finding, the current study will investigate the effects of different injection pressures (180, 210, 240 bar), on the combustion, performance and emissions of a blend of B20 blended with 50ppm of carbon nanotubes. The purpose of the current research is to determine whether there are synergies between the optimal injection pressure and the fuel blends with additives in order to maximize the engine’s efficiency while minimizing emissions.

Despite extensive research on biodiesel and injection pressure effects, research on waste cooking oil biodiesel blends augmented with advanced nanomaterials such as graphene and CNTs remains scarce. Accordingly, the present investigation aims to analyze the synergistic effects of different injection pressure levels and the incorporation of graphene and carbon nanotubes (CNTs) on the combustion dynamics, performance metrics, and emission profiles of waste cooking oil biodiesel blends in a compression ignition engine. The present investigation constitutes an initial attempt to bridge this unexplored area in the literature, providing novel insights into improving fuel atomization, combustion stability, and pollutant reduction. By exploring the synergistic effects of injection pressure optimization alongside nanoparticle additives, this study advances the formulation of eco-efficient and high-performance biodiesel fuels tailored for application in diesel engine systems.

## Resources, tools, and procedures

### Production of biodiesel from waste cooking oil

Table 1 presents the physicochemical properties of raw waste cooking oil and its biodiesel (WCO-B100) in comparison with ASTM D6751 and EN 14214 standards, showing that transesterification significantly improves key fuel properties such as viscosity, acid value, and cetane number, bringing them within acceptable limits for diesel engine applications. The primary source of waste cooking oil was the Sathyabama University Cafeteria. The spent oil from the repeated thermal cycles was filtered by removing all food debris and small particles. The oil was then heated to 120 °C to remove excess moisture to ensure no free water would interfere with transesterification. The free fatty acid (FFA) concentration was measured by titrating with 0.2 N sodium hydroxide using phenolphthalein indicator to determine accurately the amount of catalyst needed for transesterification. With the FFA concentration known, the pre-treated oil was mixed with methanol at a molar ratio of 5:1 in the presence of 1 wt% potassium hydroxide. The mixture was then stirred magnetically at 70 °C for seven hours. The product was left to settle for 24 h, then the bottom glycerol rich layer was separated and discarded. The top methyl ester layer was then washed with warm distilled water until it was free of residual catalyst, soaps, and methanol. Once free of contaminants, the pure biodiesel was dried and produced a yield of about 90%. Using the mass balance of the initial mass of waste cooking oil and the final mass of the purified methyl esters, the yield of biodiesel was estimated to be about 90%. Based on the specifications of ASTM D-6751, only the top methyl ester layer after cleaning and drying was to be considered when determining the yield of biodiesel. Following completion of transesterification, the reaction mixture was placed into a separatory funnel and left to settle for 24 h. The lower glycerol layer was then poured off and the top methyl ester layer was washed with warm distilled water until the wash water was clear; this indicated the removal of residual catalysts and soaps. The clean biodiesel was then dried at 70 °C to remove any remaining moisture. 

**Table 1:** Properties of raw waste cooking oil and its biodiesel.

Property	Raw waste cooking oil	Biodiesel (WCO-B100)	ASTM D6751 standard	EN 14214 standard
Density @ 15 °C (g/cm³)	0.918	0.868	0.86–0.90	0.86–0.90
Kinematic VISCOSity @ 40 °C (mm²/s)	35.2	4.6	1.9–6.0	3.5–5.0
Flash point (°C)	274	168	≥ 130	≥ 120
Pour point (°C)	−2	−6	Report	Report
Cloud point (°C)	3	1	Report	Report
Calorific value (MJ/kg)	38.2	40.5	–	–
Acid value (mg KOH/g)	6.2	0.38	≤ 0.5	≤ 0.5
Cetane number	43	53	≥ 47	≥ 51

### Physicochemical characterization of graphene and CNT nanoparticles

The graphene and carbon nanotube (CNT) nanoparticles obtained from Sigma-Aldrich (India) were structurally and physically characterized to assess their physical, chemical, and surface characteristics prior to being incorporated in the fuel; the specific surface area of the graphene was 30–50 m²/g and its lateral size was approximately 20 nm while the surface area of the CNTs exceeded that of graphene by over 5 times; they had diameters of about 20 nm and purities of 97% and 98% respectively-Ray diffraction (using PANalytical X’Pert PRO) confirmed the graphitic character of both types of material and identified crystalline phases within each type of particle. Morphology was assessed through use of a FEI Tecnai G2 transmission electron microscope, wherein at least 125 particles per sample were analyzed so that the statistical reliability of the particle size distributions could be ensured. The specific surface areas of the samples were assessed via nitrogen adsorption (by BET), after samples were degassed for 3 h at 105 °C; triplicate assessments were conducted on each sample so that the reproducibility of the assessments would be ensured. Understanding how these nanoparticle structures, sizes and surfaces affect both fuel improvement and the combustion process was highly dependent upon this detail physicochemical characterization.

### Morphological and structural characterization of graphene and CNT

**Fig. 1:**
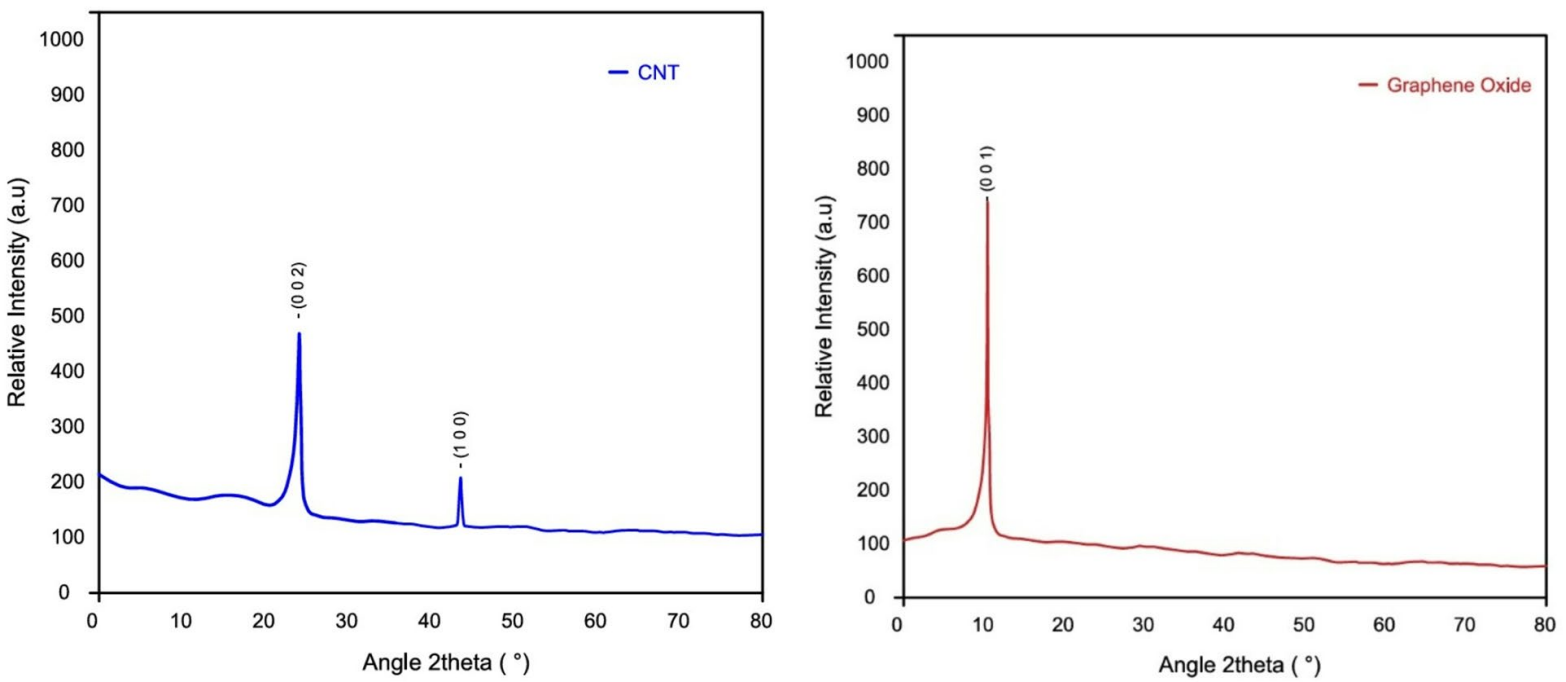
XRD images of CNT and graphene nanoparticles.

**Fig. 2:**
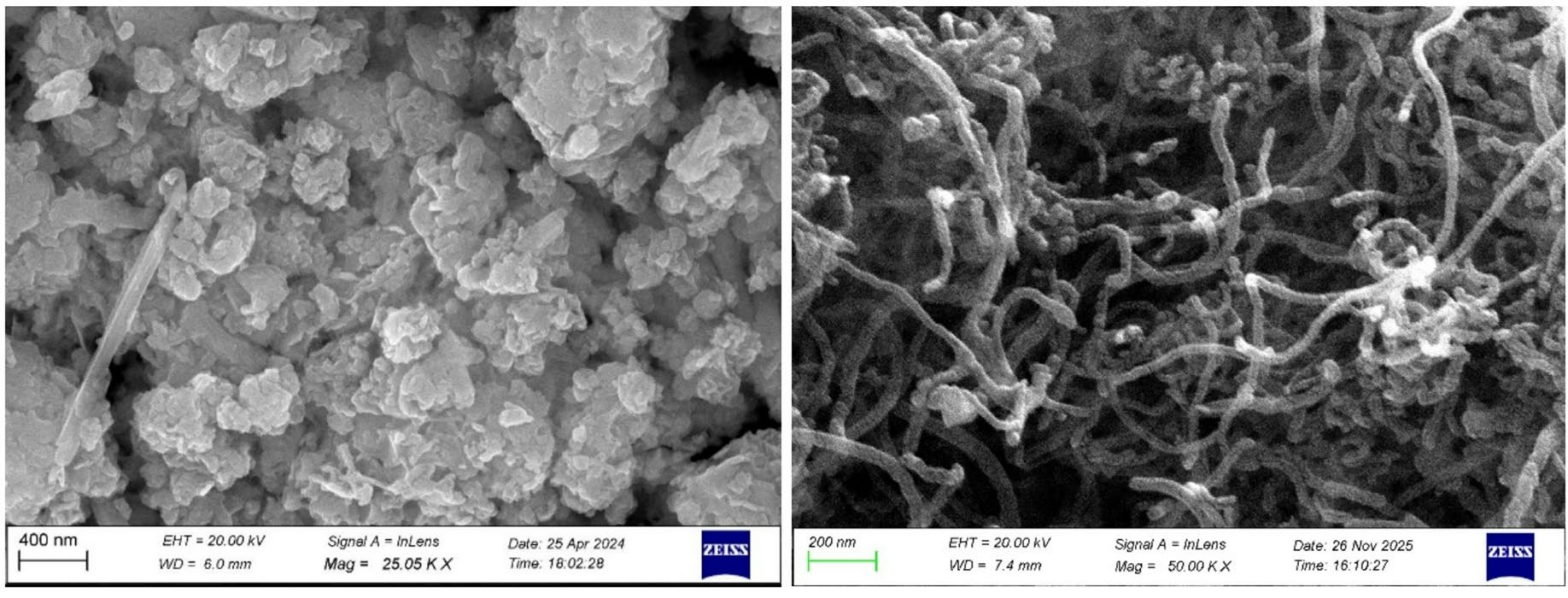
SEM images of graphene and CNT nanoparticles.

The structural and morphological characteristics of the carbon nanotubes (CNTs), produced via synthesis, were investigated using x-ray diffraction (XRD) and scanning electron microscopy (SEM). The XRD pattern (Fig. 1) provides evidence of the crystalline nature of the CNTs; whereas, the SEM images provide additional detail related to the surface morphology and tube structure of the CNTs. The presence of a distinct peak for 2θ = 26° as well as two peaks corresponding to the (002) and (100) planes of Graphene, respectively, indicate a high level of crystallinity and the presence of interlayer spacings of approximately 0.34 nm. The presence of two peaks for the (002) and (100) planes validates the Graphene -like nature of the material. The amplitudes of the peaks observed also reflect the size scales and the potential stacking defects of the materials. The SEM analysis (Fig. 2) of the CNTs provided evidence of the characteristic entanglement and tubular morphology of CNTs; the high aspect ratio and smooth surface morphology of the CNTs indicate the successful production of CNTs with suitable structural and morphological integrity. The attributes of the CNTs utilized in this research are provided in Table 2; these include information on the physical dimensions, purity and source of the CNTs. These attributes are important in fuel-related applications since they significantly impact dispersion behavior, reactivity and enhanced performance.

**Table 2:** Nanoparticle characteristics in the present investigation.

Category	Purity	Colour	SSA
Graphene	+ 97%	Yellow	30-50 m^2^/g
CNT	+ 98%	Dark yellow	> 128 m^2^/g

### Preparation and stabilization of nanoparticle-infused biodiesel blends

Following the characterization, both graphene and CNT nanoparticles were added into the B20 diesel-biodiesel blend separately. In order to determine the appropriate nanoparticle concentration to add to the B20 blends, a preliminary screening study was conducted along with corroborating studies from literature. Based on these studies, a fixed concentration of 50 ppm was chosen as the appropriate concentration for adding nanoparticles to each B20 blend. Studies conducted to examine the effects of adding nanoparticles at concentrations of 25, 50 and 75 ppm to the B20 diesel-biodiesel blend demonstrated that a concentration of 50 ppm would provide the best balance of maintaining sufficient dispersion stability, enhancing combustion and maintaining injector safe viscosity. Adding more than 50 ppm of nanoparticles increased the tendency for the particles to agglomerate and increased the viscosity of the blends. These increases may have affected the spray characteristics of the blends. Additionally, this concentration has been shown in other literature to be an optimal concentration range for the addition of nanoparticles to fuels. An example of literature supporting the use of 50 ppm as the optimal concentration range is a study conducted by Bidir et al.^32^. In their study they demonstrated that the optimal concentration range for the addition of nanoparticles to fuels was 40–55 ppm. SDS (Sodium Dodecyl Sulfate), was used as a dispersant due to its strong ability to disperse carbon-based nano-materials. Both CNTs and graphene are prone to agglomerating due to their high surface energies and van der Waal forces. By adsorbing onto the surface of the nanoparticles, SDS imparts electrostatic repulsion to the particles resulting in a lack of particle-particle attraction and allowing for long term suspension stability. In addition to providing electrostatic repulsion, the hydrophobic tail of SDS provides improved wettability and uniformity of nanoparticle distribution throughout the biodiesel-diesel blend during ultrasonic treatment. Long term consistency of combustion and emissions are critical to engine testing and therefore it is essential to maintain a stabilized nanoparticle dispersion throughout the time period in which the blends will be tested. Each B20 blend contained one type of nanoparticle; either graphene or CNTs. No blended additive samples were made. Each B20 blend containing a nanoparticle was placed in airtight opaque glass containers in order to prevent oxidation and contamination until engine testing. Nanoparticle dispersion was performed using a probe-type ultrasonicator (Model: Sonics Vibra-Cell VCX750) operating at a frequency of 20 kHz and 60% amplitude, delivering a maximum power of 750 W. The physicochemical properties of diesel, biodiesel, and nano-additive blended fuels were determined using standard ASTM testing methods, and the results are presented in Table 3.

**Table 3:** Physicochemical properties of nano-additive blended fuels.

Fuel sample	Density (kg/m³)	Kinematic viscosity (mm²/s @40°C)	Calorific value (MJ/kg)	Flash point (°C)	Cetane number	ASTM method
B20 (20% biodiesel + 80% diesel)	842	3.1	42.3	72	49	ASTM D4052/D445
B20 + Graphene	845	3.15	42.6	74	50	ASTM D4052/D445
B20 + CNT	847	3.18	42.8	75	51	ASTM D4052/D445

### Engine setup and testing procedure

**Fig. 3:**
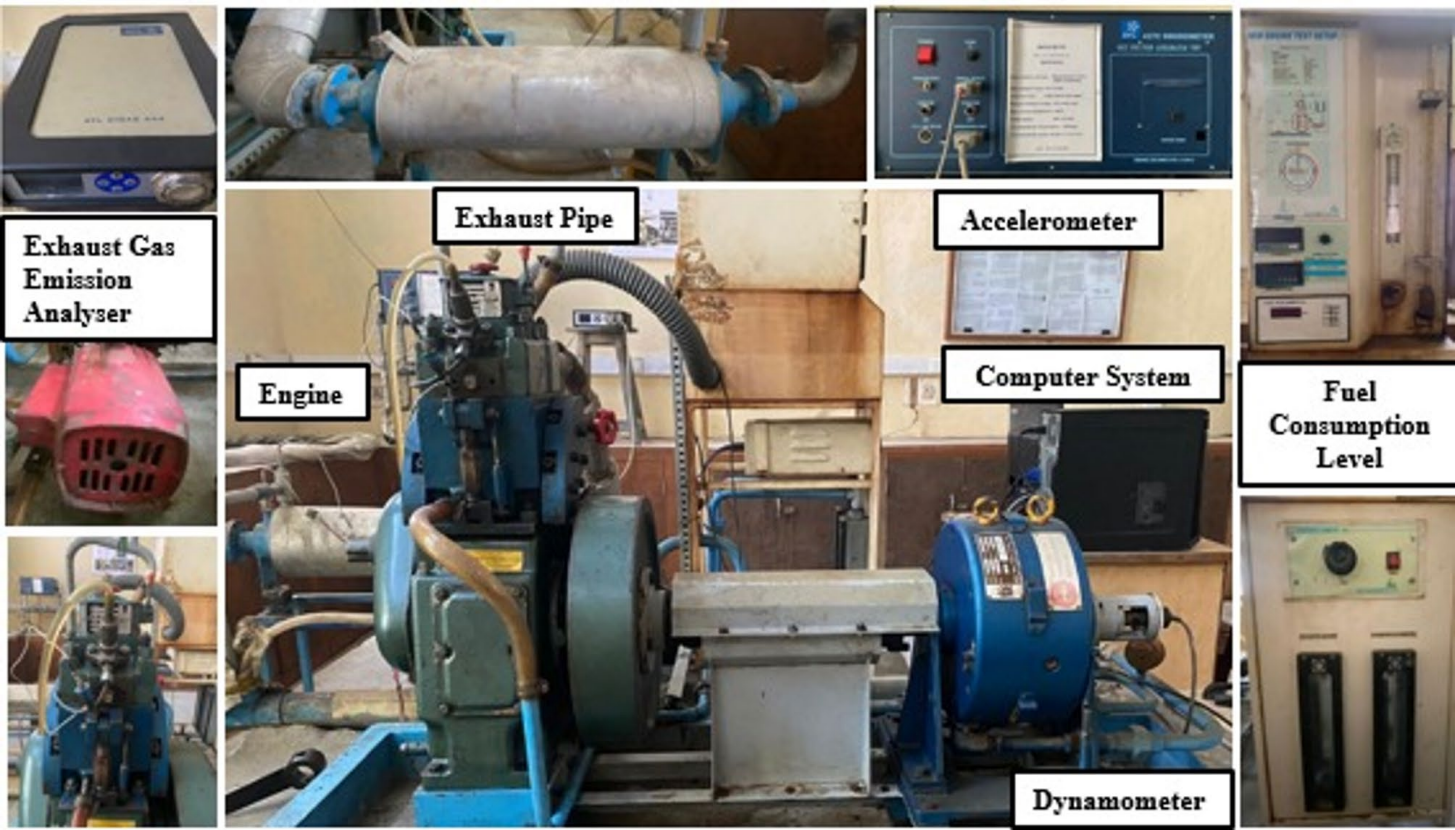
Engine photograph from the test rig^33^.

The fuel characteristics and performance and emissions characteristics of the prepared nano-blends were examined utilizing a single cylinder, 4 stroke, air cooled Kirloskar Diesel Engine with direct injection. The diesel engine ran at a constant speed of 2000 RPM with a maximum power output of 4.4 KW and a compression ratio of 17.5:1. The diesel engine utilized a solenoid actuated injector with a three-hole nozzle and had a timed injection of 23° bTDC. To determine how the performance and emissions characteristics of the diesel engine would be affected by the injection pressure of the fuels; a series of experiments were conducted at injection pressures of 180, 210 and 240 bar. The engine load was controlled using an eddy current dynamometer and was varied at loads of 25%, 50% 75% and full load. The in-cylinder pressure was determined utilizing a piezoelectric pressure sensor that was synchronized with a crank angle encoder for the purpose of accurately determining the heat release rate from the combustion process. Emissions of CO, HC, NOx, and CO_2_ were measured utilizing an AVL Digas 444 analyzer and smoke opacity was measured utilizing an AVL smoke meter. Prior to conducting the tests all measuring devices were properly calibrated to ensure that the uncertainty of each measurement device did not exceed ± 2%. The physical properties of the fuels were determined utilizing the American Society for Testing and Materials (ASTM) standard D6751, European Norm (EN) standard 14,214, and ASTM standard D240. The photograph of the experimental test equipment utilized to conduct these studies is presented in Fig. 3 and a description of the specifications of the Kirloskar single cylinder diesel engine are listed in Table 4.

**Table 4:** Technical specifications of the Kirloskar single-cylinder diesel engine.

Parameter	Description
Engine model	Kirloskar, single-cylinder, four-stroke, air-cooled diesel engine with direct injection
Cylinder Bore × Stroke	87.5 mm diameter × 110 mm stroke length
Number of fuel injector holes	3
Displacement volume	661 cubic centimeters (cc)
Piston head design	Hemispherical crown
Injection timing	Set at 23° before the piston reaches Top Dead Center (bTDC)
Fuel spray cone angle	120 degrees
Compression ratio	Fixed at 17.5:1
Injector nozzle diameter	0.3 mm
Intake valve timing	Opens 4.5° before Top Dead Center (bTDC), closes 35.5° after Bottom Dead Center (aBDC)
Exhaust valve timing	Opens 35.5° before Bottom Dead Center (bBDC), closes 4.5° after Top Dead Center (aTDC)
Rated power output	Approximately 4.4 kilowatts (kW)
Rated speed	2000 rpm
Engine torque	16 Nm at 2000 rev/min
Fuel injection time	24^o^ BTDC
Injection pressures used	180 bar, 210 bar, 240 bar

### Assessmet of experimental uncertainties

Evaluating the uncertainty associated with engine performance and emission data is essential for ensuring the credibility of the experimental outcomes. In this work, uncertainty values were determined based on the specifications of the measuring instruments and the adopted methodologies, in alignment with established guidelines reported in previous studies. A detailed overview of the measurement ranges, instrument accuracies, and calculated percentage uncertainties for each variable is provided in Table 4. The total experimental uncertainty was determined using the Root Sum Square (RSS) approach:

1 $${\text{Overall uncertainty }} = {\text{ }}\surd \left( {{\mathrm{U}}^{{\mathrm{2}}} _{{{\mathrm{BP}}}} + {\mathrm{U}}^{{\mathrm{2}}} _{{{\mathrm{BTE}}}} + {\mathrm{U}}^{{\mathrm{2}}} _{{{\mathrm{BSFC}}}} + {\mathrm{U}}^{{\mathrm{2}}} _{{{\mathrm{CO}}}} + {\mathrm{U}}^{{\mathrm{2}}} _{{{\mathrm{UHC}}}} + {\mathrm{U}}^{{\mathrm{2}}} _{{{\mathrm{NOx}}}} + {\mathrm{U}}^{{\mathrm{2}}} _{{{\mathrm{Smoke}}}} + {\mathrm{U}}^{{\mathrm{2}}} _{{{\mathrm{EGT}}}} } \right)$$

In this section, the symbol U denotes the uncertainty level associated with each parameter. The numerical values applied in this analysis are summarized in Table 5, yielding an overall experimental uncertainty of ± 2.1%.

**Table 5:** Gadget datasheet with uncertainty numbers.

Devices	Accuracy level	Range	%uncertainties
Gas analyzer	± 0.02% ± 0.02%	CO 0–10% CO_2_ 0–20%	± 0.1 ± 0.10
Smoke meter	± 0.1	HSU 0–100	± 1.0
Temperature indicator	± 1 °C	0–1100 °C	± 0.1
Stopwatch (digital)	± 0.1s	-	± 0.2
Pressure sensor	± 1 bar	0–120 bar	± 0.1
Cranking angle encoder	± 1°	-	± 0.2
Speed sensor (proximity type)	± 10 rpm	0–1000 rpm	± 1.0
Torque indicator	± 0.1 N m	0–100 Nm	± 0.1

### Performance parameters

The dynamometer used to measure engine output consists of electromagnets that generate a magnetic field in which the rotors, connected flexibly to the crankshaft, rotate. Variations in the electric current flowing through the electromagnets adjust the magnetic resistance, which in turn applies a controllable load to the engine. Torque (T) is determined by multiplying the reactive force (F) measured by a strain gauge with the lever arm distance (R) from the pivot point. This arrangement enables precise detection of the mechanical load imposed on the engine. Once torque is known and the engine speed (N) is maintained constant, the brake power (BP) can be calculated

2 $${\text{T }} = {\text{ F}} \times {\mathrm{R}} \quad \left( {{\mathrm{Nm}}} \right)$$

3 $${\text{BP }} = \frac{{2\pi NTS}}{{60}}\quad \left( {{\mathrm{kW}}} \right)$$

The ‘S’ represents the dynamometer reading. Braking Power (BP) is calculated using Eq. (3), which accounts for varying load conditions ranging from 0% to 100%, thereby reflecting the engine’s power output under different operational loads. while maintaining an engine speed of one thousand revolutions per minute (rpm). Using an analog stopwatch to record the time it takes to consume a given amount of 10 cc of gasoline, as shown in Eq. (4), is the standard method for measuring fuel consumption.

4 $${\text{TFC }} = \frac{{\rho \times V}}{t}\quad \left( {{\mathrm{g}}/{\mathrm{s}}} \right)$$

In this context, TFC represents the total fuel consumption, while ρ denotes the fuel density. The variable v corresponds to the volume of fuel consumed, and t indicates the time recorded by the stopwatch for the consumption of 10 cc of fuel. (BSFC) is calculated by relating TFC to the (BP)of the engine. Furthermore, (BTE) is evaluated using Eq. (5), which incorporates the energy input and the useful mechanical output.

5 $$\text {BTE} = \:\frac{BP}{TFC\times\:CV}\:\times\:100\:\:\:\:\:\left(\%\right)$$

‘C_V_’ denotes the calorific value of the fuel being analyzed, indicating the total amount of energy liberated upon its complete combustion. The air intake flow rate, a critical parameter for evaluating engine performance, is measured precisely using a U-tube manometer. This device is connected to an orifice with a diameter of approximately 20 mm, which creates a pressure differential proportional to the airflow. The volumetric flow rate of air (V_A_​) is then calculated using Eq. (6), which considers the pressure difference indicated by the manometer, the orifice area, and fluid flow characteristics. Accurate measurement of the airflow is essential to determine the air-fuel ratio, combustion efficiency, and overall engine performance.

6 $${\mathrm{V}}_{{\mathrm{A}}} = {\text{ C}}_{{\mathrm{d}}} \times {\mathrm{A}} \times \surd \left( {2gH} \right)~\quad \left( {{\mathrm{m}}^{{\mathrm{3}}} } \right)$$

In this setup, the discharge coefficient (Cd) is assumed to be 0.60, representing the flow behavior through the orifice. The parameter A refers to the orifice’s cross-sectional area (in m²), which significantly affects the volumetric airflow rate. The variable H denotes the height of the water column in the U-tube manometer, measured in meters (m), and serves as an indicator of the pressure differential driving the flow. Lastly, g represents the acceleration due to gravity (9.81 m/s²), a fundamental constant used in fluid dynamics calculations to determine the rate of airflow through the orifice.

## Findings and discussion

### Effect of injection pressure on performance and emissions

The main goal of this study was to investigate the influence of injection pressure on combustion performance and emission of a blend of diesel and biodiesel with the addition of graphene and carbon nanotubes. The experimental tests were performed using three different injection pressures (180, 210, and 240 bar) of the aforementioned blends and the results were analyzed. Increasing the injection pressure improves the breakup of the fuel droplets resulting in better air-fuel mixing inside the combustion chamber. The enhanced breakup of the fuel droplet has a direct positive impact on the rate of evaporation of the fuel and subsequently enhances the overall combustion efficiency. The engine performance and emissions are directly affected by the combustion efficiency and therefore increasing the injection pressure will have a significant impact on both the engine performance and the engine emissions. The best engine performance among the examined cases was achieved with the 240-bar injection pressure based on the highest values of brake thermal efficiency and lowest values of brake specific fuel consumption. The reason behind this is the fact that the injection pressure was the highest among all the examined cases. Because of the complete oxidation of the fuel during the combustion process the emissions of carbon monoxide, hydrocarbons and smoke were reduced due to the improved combustion efficiency with increased injection pressure. The data related to the engine performance and emissions for the studied cases at different injection pressures are provided in Table 6. Table 6 shows how increasing the injection pressure can enhance the combustion efficiency and fuel atomization, thus improving the Brake Thermal Efficiency (BTE), reducing the emissions of BSFC, CO and HC but increasing the emissions of NOx slightly, due to the higher combustion temperatures. Finally, it should be noted that the best performance and emission characteristics were obtained by W20G3 and W20C3 among all the tested blends.

**Table 6:** Effect of injection pressure on engine performance and emission characteristics at full load.

Injection Pressure (bar)	Blend	BTE (%)	BSFC (kg/kWh)	CO (%)	HC (ppm)	NOx (ppm)
180	B20	29.2	0.315	0.052	54	820
180	W20G3	30.5	0.303	0.047	49	845
180	W20C3	30.9	0.298	0.045	47	858
210	B20	30.6	0.300	0.047	50	885
210	W20G3	32.1	0.287	0.041	44	912
210	W20C3	32.6	0.282	0.039	42	925
240	B20	31.8	0.286	0.043	46	930
240	W20G3	33.4	0.272	0.036	39	956
240	W20C3	34.0	0.267	0.034	37	970

### Influence of injection pressure on brake thermal efficiency

**Fig. 4:**
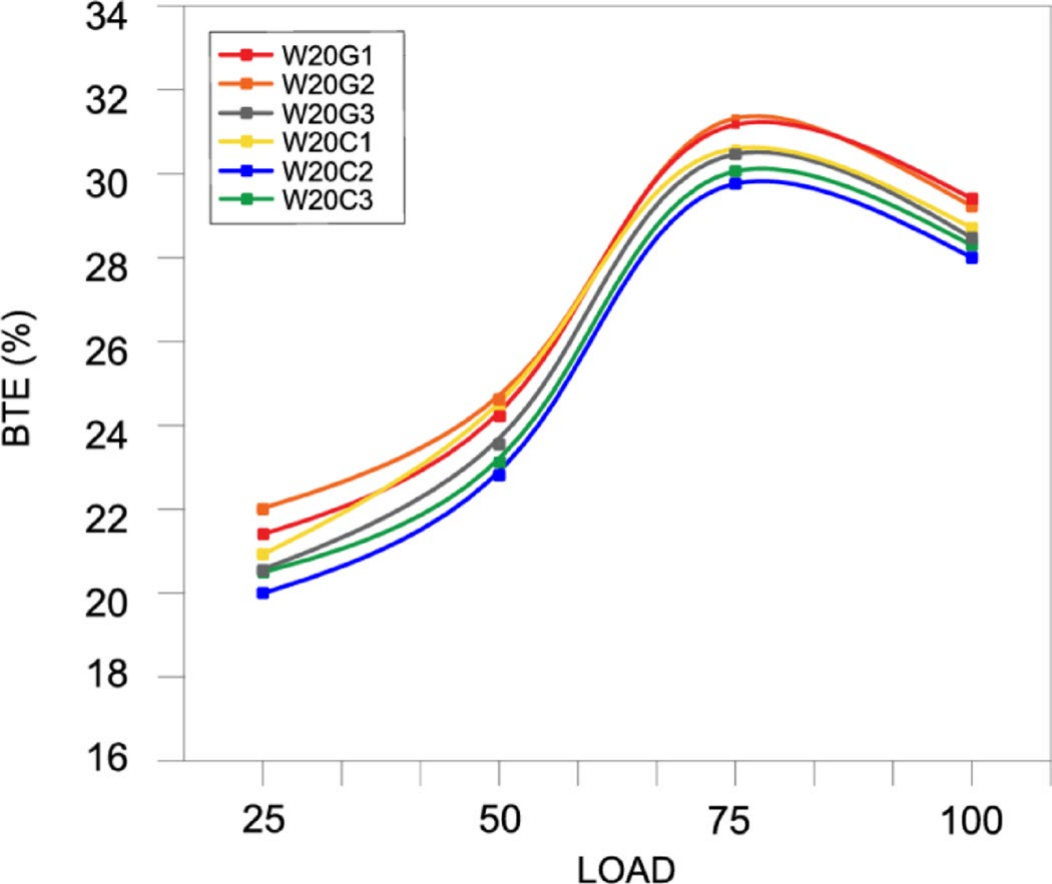
Variation of brake thermal efficiency (BTE) with Engine Load.

In Fig. 4, the relationship among brake thermal efficiency (BTE) for the various graphene- and carbon nanotube (CNT)-enhanced blends of biodiesel (W20G1, W20G2, W20G3, W20C1, W20C2, and W20C3) at engine loads of 25% to 100% and injection pressures of 180 to 240 bar are presented. With increasing load, it is evident that all blends have a substantial rise in BTE, due primarily to increased cylinder temperature, improved fuel vaporization, and better mixing between the fuel and air. For the 25% load, the BTE values are in the lower range (20–22%) as a result of decreased oxidation and turbulence. In contrast, as the load increases, the combustion process intensifies and peak BTE values of 29–32% are achieved approximately 75% of the way up to full load. Significant improvements in BTE are observed when the injection pressure is raised from 180 to 240 bar, where an average gain of 1.7–2.0.7.0% across the blends is found. Increased injection pressure results in smaller fuel droplet sizes, increases the spray cone angle, and enhances atomization. As such, a more uniform fuel-air mixture is obtained, which is supported by earlier research conducted by Kumar et al.^34^. who concluded that higher injection pressure resulted in reduced ignition delays and accelerated premixed combustion heat release. Additionally, Seman et al.^35^. showed that BTE was significantly increased at pressures above 210 bar, primarily as a result of improved spray dynamics. Furthermore, Banerjee et al.^23^. and Polat et al.^36^. demonstrated that the combination of nano-additives and increased injection pressures produced a large degree of improvement in combustion efficiency in compression ignition (CI) engines. Between the two particle types, the graphene-based blends consistently exhibited a higher BTE than the CNT-based blends at each load and pressure condition tested. Graphene has been shown to have superior thermal conductivity, a larger reactive surface area, and high oxygen availability that synergistically act to enhance combustion kinetics and increase the rate of heat liberation. Such characteristics result in a shorter ignition delay, improved oxidation of intermediate products, and reduced cycle-to-cycle variability. The W20G3 blend exhibited the highest BTE for all test conditions, indicating the strong catalytic nature of graphene, as was previously reported by Vadapalli et al.^37^. and Islam et al.^38^. Graphene was demonstrated to improve heat transfer, enhance micro-explosion behavior, and promote nearly complete combustion. CNT-based blends (W20C1, W20C2, W20C3) also improved BTE over base biodiesel due to their ability to create in-cylinder turbulence and increase thermal conductivity. The tubular structure of CNT provides better fuel break-up; however, CNT exhibits slightly lower catalytic reactivity than graphene, resulting in a lower thermal efficiency. Similar conclusions were reached by Rao et al.^31^ and Aziz et al.^39^, who demonstrated that CNT improved combustion but was less effective than oxygen-rich nanomaterials. At full load, a small decrease in BTE is seen for all blends, primarily due to the presence of fuel-rich zones, increased viscosity of the nanoparticle-infused biodiesel, and increased thermal and mechanical losses. Similar conclusions were made by Zheng et al.^40^. who showed a minimal drop in efficiency at maximum fueling rates, while demonstrating improved atomization. Overall, these results demonstrate that nanoparticle-assisted B20 blends, specifically those containing graphene, combined with increased injection pressure (240 bar), produce the greatest improvements in combustion efficiency through improved atomization, rapid evaporation, catalytic oxidation, and more efficient use of the fuel’s chemical energy.

### Analysis of BSEC in biodiesel-nanoparticle blends

**Fig. 5:**
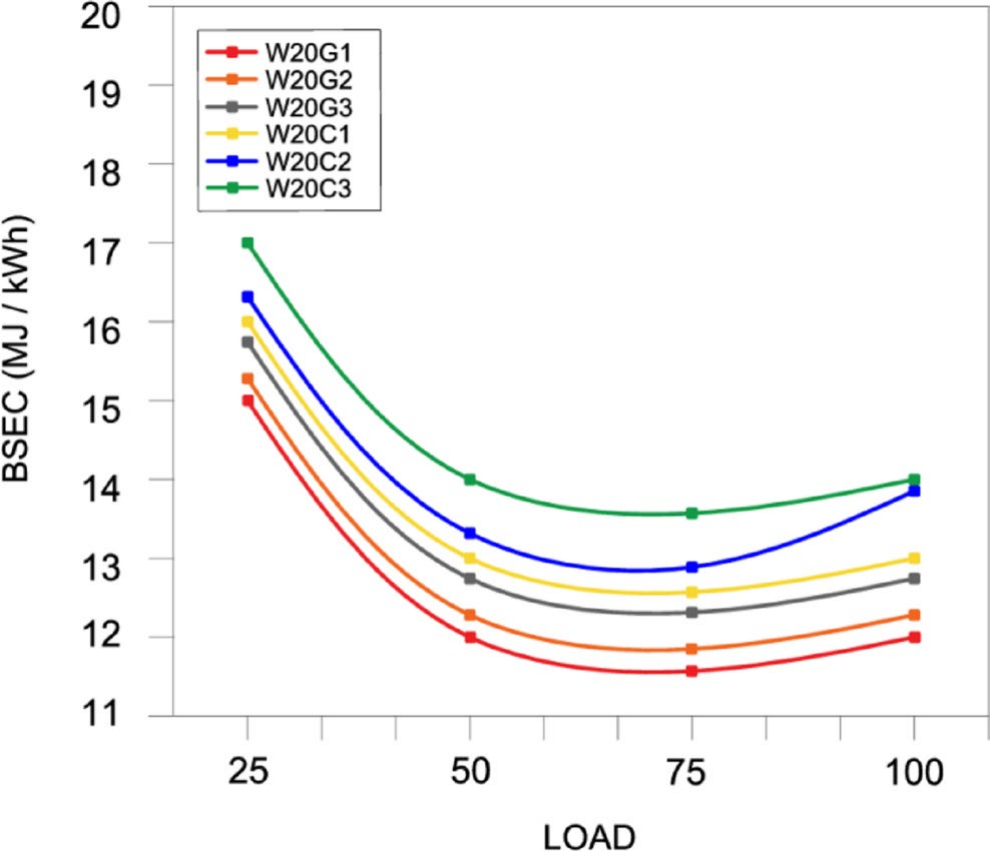
Variation of brake specific energy consumption (BSEC) with engine load.

The Fig. 5 illustrates how the Brake Specific Energy Consumption (BSEC), or the amount of energy consumed to produce one kilowatt-hour of braking power, varies with the amount of nanoparticles added to the B20 bio-diesel blend. The BSEC was measured under various engine loadings (25–100%) and fuel injection pressures (180–240 bar). Generally speaking, there exists a strong correlation between the engine loading and the BSEC. As the engine loading increases, the BSEC decreases, reaching a minimum at approximately 75% load. This relationship is generally consistent throughout the literature cited in this study and is primarily due to the increased temperature and combustion homogeneity at elevated engine loads, which allow for a reduction in the amount of energy necessary to produce the same amount of braking power. According to Sazhin et al.^41^ and Ghojel et al.^42^, it has been previously shown that the addition of nanoparticles to biodiesel results in a reduction in BSEC, primarily due to the enhanced micro-explosion characteristics and catalytic surface reactions of the particles within the combustion process. In general, all three types of nanoparticle additives (Graphene and Carbon Nanotubes) produced reductions in BSEC when compared to the base B20 bio-diesel. However, the graphene based blends (W20G1, W20G2, W20G3) exhibited the lowest BSEC values at all operating conditions examined. Specifically, W20G3, exhibited the lowest BSEC value of approximately 11.8 MJ/kWh at 75% engine load. The improvements in BSEC exhibited by the graphene blends can be directly correlated to their ability to improve the atomization quality of the fuel and facilitate faster oxidation processes during the combustion process. Both factors are directly related to the unique properties of the graphene particle used in these studies. Specifically, the extremely high thermal conductivity of the graphene particle allows for improved heat transfer rates and the unique laminar sheet morphology of the particle facilitates improved fuel-air mixing prior to combustion. Both of these factors result in improved combustion efficiencies, and thus a reduction in the amount of energy required to produce the same amount of braking power. Additionally, according to Rao et al.^43^ the use of graphene particles to facilitate the combustion process will result in improved fuel consumption rates and improved oxidation rates due to the catalytic nature of the particles. Similar to the results obtained with the graphene blends, the carbon nanotube blended fuels (W20C1, W20C2, W20C3) also exhibited improved energy efficiencies relative to the base B20 biodiesel. However, the BSEC values for the carbon nanotube blends were slightly higher than those of the corresponding graphene blends. While the tubular structure of the carbon nanotubes does contribute to the improved turbulence intensities and droplet break-up characteristics of the fuel, their catalytic activity and oxygen availability are significantly less than that of the graphene particles. Thus, while the carbon nanotube blends do provide some improvements in BSEC, they do so at a cost of higher energy consumption per unit output. This trend has been previously identified by Azarudeen et al.^44^. A major factor contributing to the improvements in BSEC observed with all blends is the increase in fuel injection pressure from 180 to 240 bar. According to Çılğın et al.^45^ and Çelebi et al.^46^, an increase in fuel injection pressure results in improved air-fuel interactions, improved thermal efficiencies, and improved premixed combustion characteristics. Additionally, the increased fuel injection pressure results in the production of finer droplets, and the development of larger spray cone angles. All of these factors contribute to a reduction in the amount of energy required to effectively convert the fuel into usable forms. At full load, a small increase in BSEC occurs for all blends. This increase is primarily due to the increased in-cylinder temperatures, increased frictional losses, and localized areas of richer fuel-air ratios developed due to the increased amounts of fuel being injected. Despite this increase, the BSEC of both the graphene and CNT nanoparticle blends are significantly lower than that of pure biodiesel, demonstrating the effectiveness of using nanoparticle-assisted combustion techniques under all fuel injection pressures.

### Combustion profiles and their impact

**Fig. 6:**
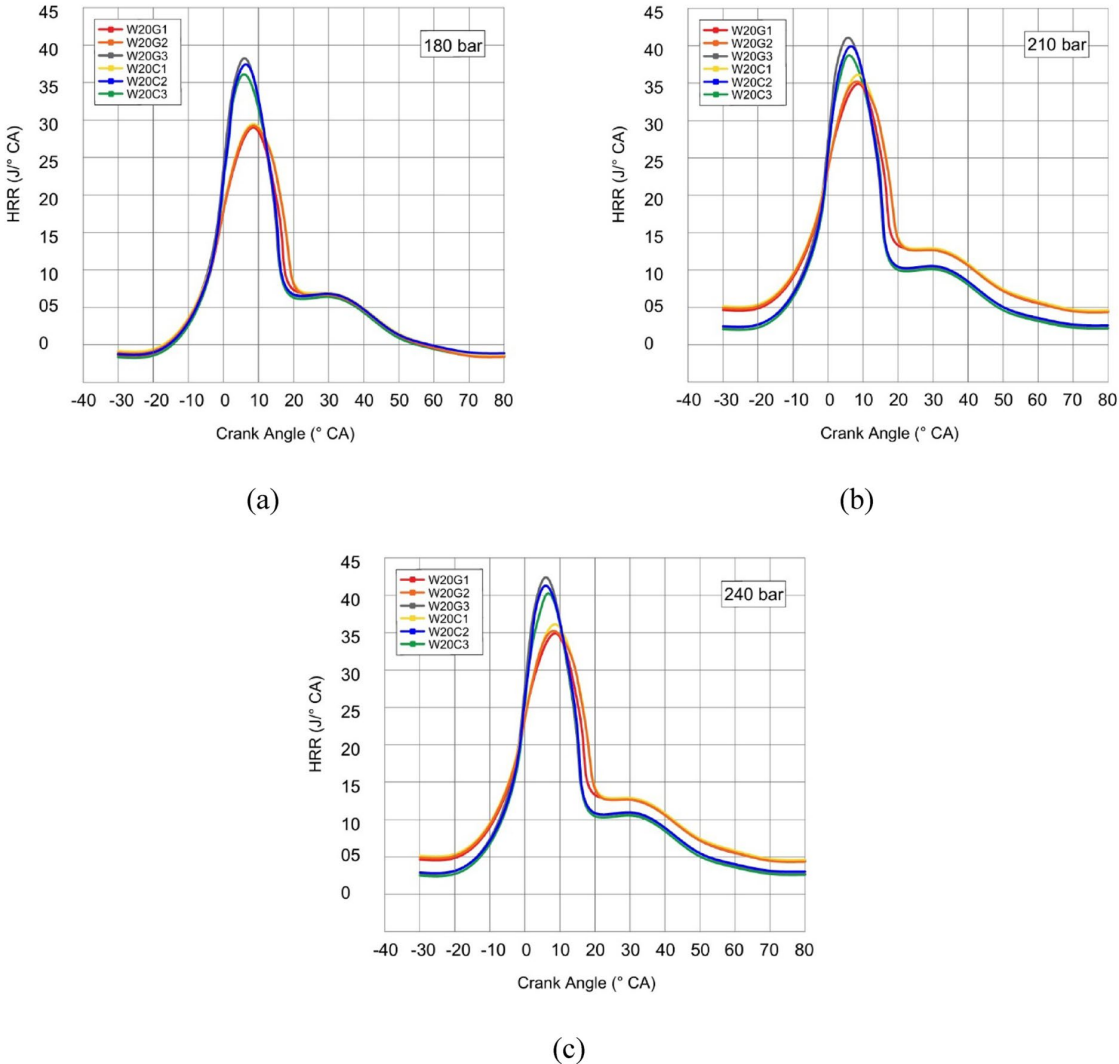
Variation of heat release rate with crank angle for different fuel blends at different injection pressures: **a** 180 bar, **b** 210 bar, and **c** 240 bar.

**Fig. 7:**
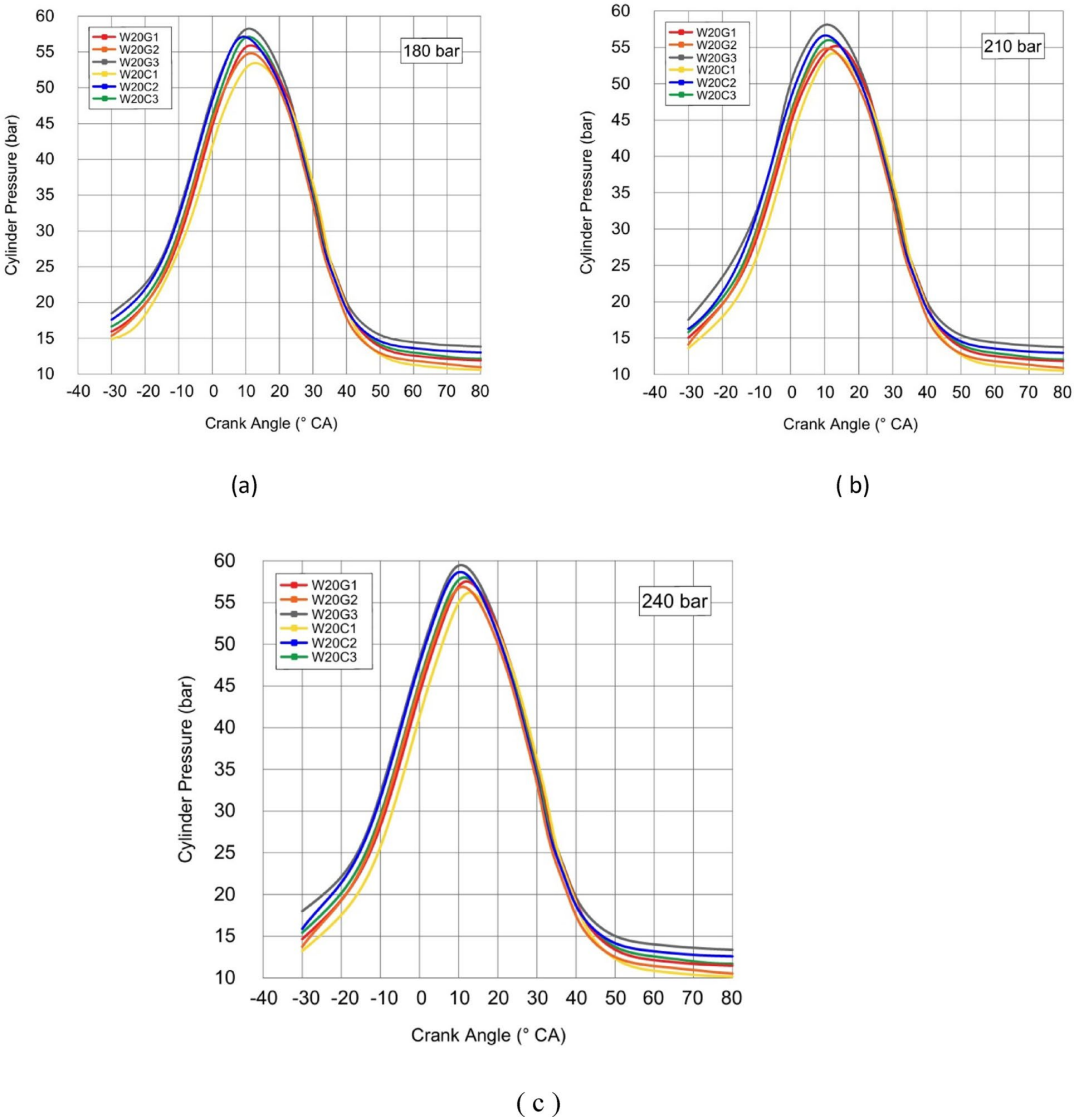
Variation of in-cylinder pressure with crank angle for different fuel blends at different injection pressures: **a** 180 bar, **b** 210 bar, and **c** 240 bar.

The HRR (Heat Release Rate) vs. Crank Angle (HRR–θ), as shown in Fig. 6, shows the variations of HRR with crank angle (θ) at different injection pressures under full load operating condition. All the tested blends show similar HRR curve patterns showing typical combustion pattern consisting of premixed combustion phase followed by diffusion controlled burning phase. Among the tested blends, HRR peaks of graphene based fuel (W20G3) are higher and sharper than those of CNT based blends. This indicates better atomization, better air-fuel mixture, and faster combustion phasing for the graphene based blends. In comparison with the results at lower injection pressures (180 bar), increasing the injection pressure to 240 bar showed significant increases in HRR for all the tested blends. Maximum HRR values obtained for the six tested blends were 30.1 J/°CA, 30.2 J/°CA, 31.24 J/°CA, 31.56 J/°CA, 36.5 J/°CA and 35.2 J/°CA for W20G1, W20C1, W20G2, W20C2, W20G3 and W20C3, respectively. For all the injection pressures, it is observed that graphene blended fuels have produced higher HRR values than CNT blended fuels, which clearly demonstrate that the graphene nanoparticles have more effective catalytic activity and better thermal conductivity. The HRR of both W20G3 and W20C3 has been increased by about 19% and 21.9% by raising the injection pressure from 180 bar to 240 bar, mainly due to improved atomization and more uniform air-fuel mixing. Figure 7 shows the in-cylinder pressure variation with crank angle (P–θ) for the same set of blends. It is evident from the figure that the injection pressure affects the peak cylinder pressure and the combustion timing. Due to better fuel atomization and premixing at higher injection pressures, the start of combustion occurs earlier and the peak cylinder pressure is also higher. At full load condition, peak in-cylinder pressures for W20G1, W20C1, W20G2, W20C2, W20G3, and W20C3 were 60 bar, 59.6 bar, 63.6 bar, 62.6 bar, 61 bar, and 60 bar, respectively. As expected, cylinder pressure increased with the increase in injection pressure from 180 bar to 240 bar due to better spray atomization and combustion efficiency. However, too high injection pressure could lead to over atomization and excessive spray penetration, which could be detrimental to the combustion stability and the mechanical stresses in the engine components. Graphene enhanced blends produce approximately 1.8% higher peak in-cylinder pressure than CNT blended fuels^47,48^.

**Fig. 8:**
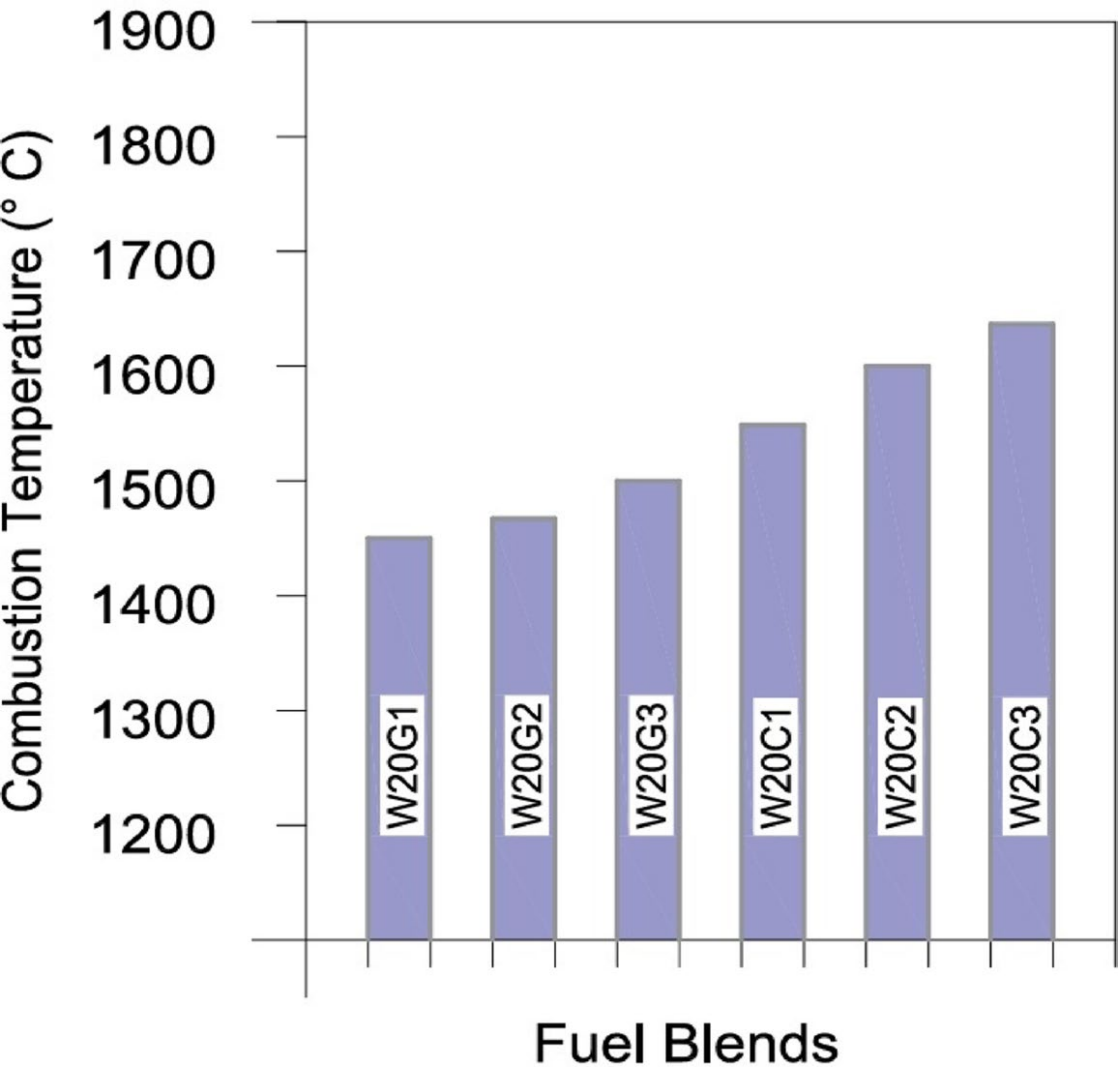
Mean combustion temperature of different nano-enhanced B20 fuel blends.

Figure 8 illustrates the mean Combustion temperature (MCT) for each fuel blend under full-load conditions. MCT is an important indicator of combustion quality, engine efficiency, and emission behavior. Higher MCT values generally reflect stronger combustion due to greater heat release inside the cylinder. However, excessively high temperatures can promote thermal NOx formation; therefore, controlling MCT through optimized injection pressure is essential. Increasing injection pressure enhances fuel atomization, producing finer droplets that burn more rapidly and elevate cylinder temperature. At 180 bar, the MCT values for W20G1 and W20C1 were approximately 1400 °C and 1450 °C, respectively. When the pressure was raised to 210 bar, the temperatures increased by about 50 °C for W20G1 and 60 °C for W20C1.Nanoparticles also influence Combustion temperature. Although graphene-based blends (e.g., W20G1) achieved higher brake thermal efficiency, they exhibited lower MGT than CNT-based blends. This indicates that graphene supports efficient combustion while moderating peak temperatures, which may help limit NOx formation. Therefore, when formulating biodiesel–nanoparticle blends, it is important to balance combustion improvement with temperature control to achieve both high performance and reduced emissions^49,50^.

### Determinants of emissions

#### Exposure of hydrocarbons

**Fig. 9:**
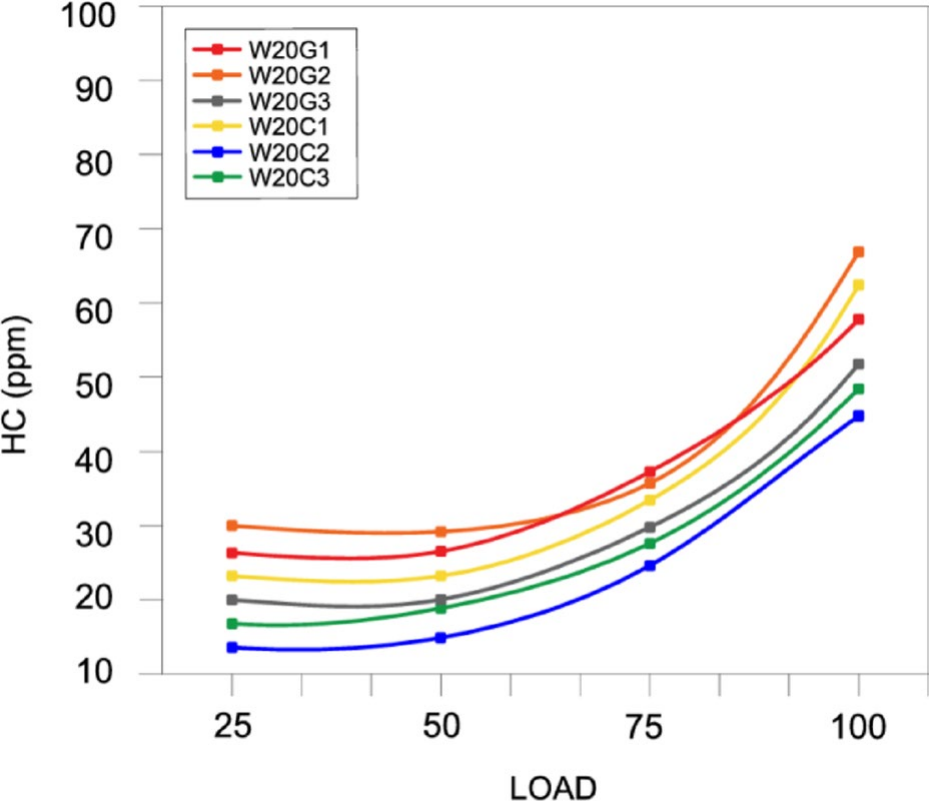
Variation of hydrocarbon (HC) emissions with engine load.

Nanoparticles improve fuel oxidation by providing a large surface area and catalytic sites that promote more complete combustion. As shown in Fig. 9, HC emissions decrease steadily with increasing injection pressure because finer fuel droplets burn more efficiently and leave fewer unburned hydrocarbons in the exhaust^51,52^. At 180 bar, the highest HC value was observed for W20G1 (50 ppm). When the injection pressure increased to 240 bar, the graphene-based W20G3 blend recorded the lowest HC level of 16 ppm. This reduction demonstrates the direct benefit of higher injection pressure in strengthening air–fuel mixing and accelerating oxidation. Graphene blends consistently produced lower HC emissions than CNT blends. This advantage is attributed to graphene’s high thermal conductivity and better oxygen availability, which support faster and more uniform combustion. At full load and 240 bar, W20G3 showed about 9% lower HC emissions compared with the CNT-based W20C3, confirming graphene’s superior catalytic role. Engine load also influenced HC formation. At 25% load, the HC emissions for blends W20G1, W20C1, W20G2, W20C2, W20G3, and W20C3 were 26, 29, 21, 23, 16, and 18 ppm, respectively. At full load, these values rose to 45, 57, 64, 48, 59, and 54 ppm due to fuel-rich zones and reduced oxygen availability. Overall, the results confirm that both higher injection pressure and nanoparticle addition especially graphene contribute to lower HC emissions by enhancing combustion completeness. This indicates that graphene-enhanced biodiesel blends offer a cleaner and more efficient alternative for CI engine applications^53,54^.

#### Variation of NOₓ emissions with operating parameters

**Fig. 10:**
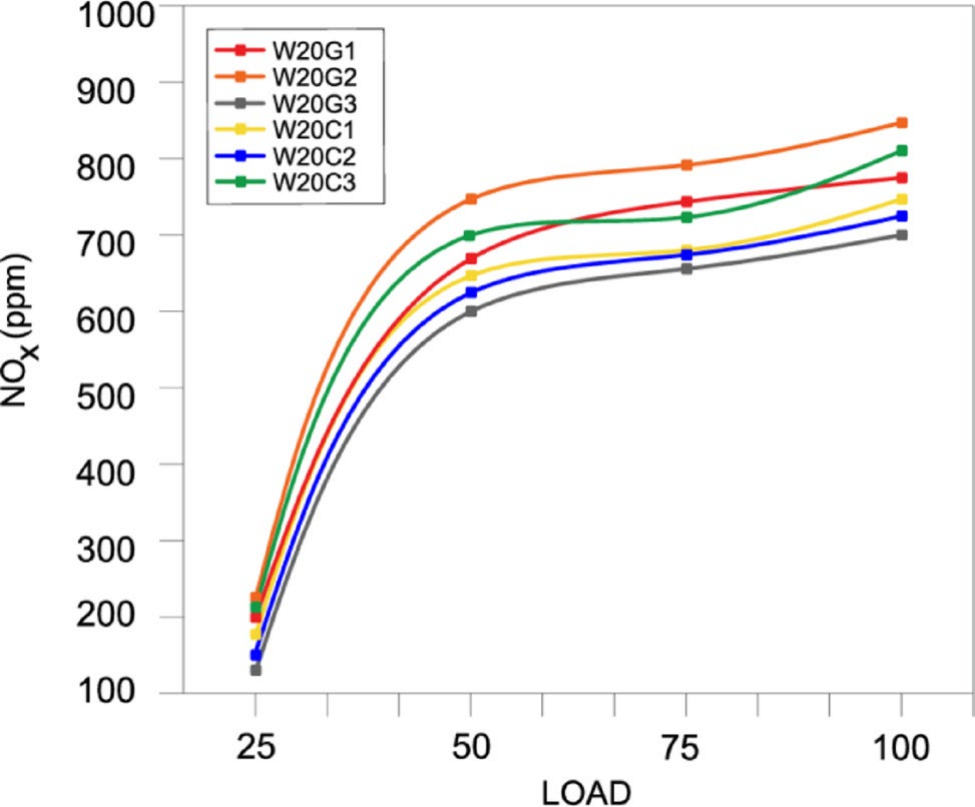
Effect of injection pressure on NOx emission.

Figure 10 presents the NOx emission levels for all nanoparticle-enhanced blends under different operating conditions. At low engine load, the NOx values for W20G1, W20C1, W20G2, W20C2, W20G3, and W20C3 were 176, 194, 147, 161, 142, and 159 ppm, respectively. As the load increased, NOx emissions rose due to higher in-cylinder temperatures, which promote thermal NOx formation. This behaviour aligns with the expected temperature–NOx relationship reported in earlier studies^55,56^. Injection pressure also influenced NOx generation. Higher pressures improved atomization and combustion efficiency but simultaneously raised peak temperatures, which intensified NOx formation. For example, W20G1 recorded 805 ppm at 180 bar, while W20C3 reached 778 ppm at 240 bar. Overall, increasing injection pressure alone reduced NOx by less than 2.1%, indicating that temperature effects dominate over improved mixing. Graphene-based blends consistently produced lower NOx emissions than CNT-based blends. Although both nanoparticles enhanced combustion, graphene improved oxidation efficiency without excessively increasing peak temperature. At full load and 240 bar, the W20G3 blend recorded the lowest NOx value (762 ppm), approximately 3.5% lower than its CNT equivalent (W20C3). Across all conditions, W20G3 showed the most controlled NOx formation, with values ranging from 140 ppm at low load to 782 ppm at full load. Overall, the results confirm that higher injection pressure increases NOx due to higher combustion temperatures, despite improving overall engine performance. Incorporating graphene nanoparticles helps moderate this rise by enabling efficient combustion with better oxygen utilization and lower peak temperatures. Future work should optimize nanoparticle dosage and injection parameters and consider after-treatment options to achieve further NOx reduction.

#### Emissions of carbon monoxide (CO)

**Fig. 11:**
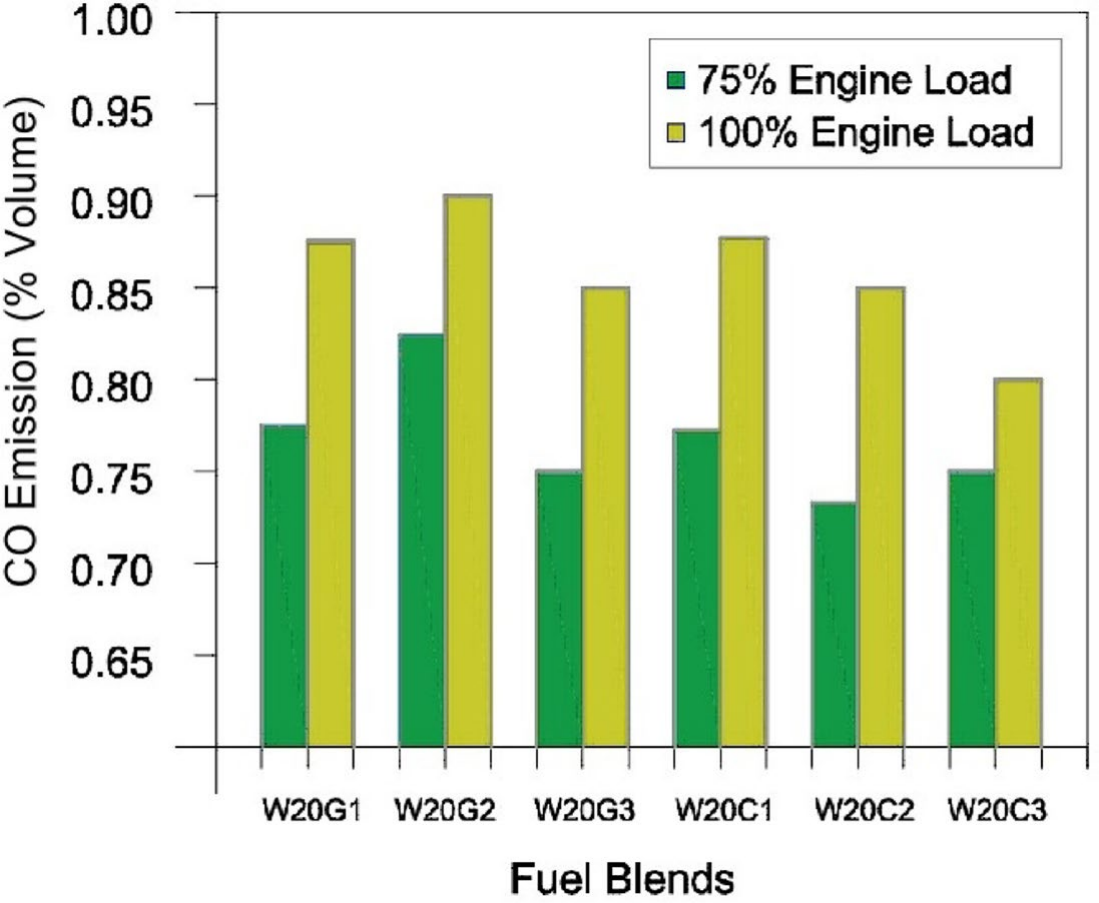
Effect of varying injection pressure levels on CO emissions.

The influence of injection pressure on the variations in CO formation is shown in Fig. 11, for the different blends tested. Combustion quality influences CO formation. In general, as injection pressure is increased, improved fuel atomization occurs, which promotes a more uniform air-fuel mixture, resulting in lower CO emissions; a factor that has been widely demonstrated through combustion research^57,58^. At 180 bar, the W20G1 blend exhibited the largest amount of CO (0.86%) of all blends tested. Increasing the injection pressure from 180 to 240 bar reduced CO for all blends tested, with the W20G3 exhibiting the least amount of CO (0.84%) of all blends tested. An increase in injection pressure from 180 to 240 bar resulted in approximately 3.8% less CO being formed when using these blends, supporting that finer spray characteristics are beneficial to oxidation efficiency. Graphene-based blends also produced lower CO emissions than CNT based blends. The reason for this is due to graphene’s greater oxygen availability and its ability to transfer heat more efficiently than CNTs, resulting in more complete combustion, therefore reducing CO formation. Graphene-based blends were approximately 1.1% lower in CO emissions than CNT based blends during high-load operating conditions. The CO emissions for the blends tested during full-load operating conditions were 0.89%, 0.87%, 0.83%, 0.84%, 0.82%, and 0.81% for W20G1, W20C1, W20G2, W20C2, W20G3, and W20C3, respectively. The lowest CO values of all blends tested occurred at 75% load, with values ranging from 0.72% to 0.76%. Therefore, it is evident that increasing the injection pressure and incorporating oxygen-rich nanoparticles (particularly graphene), have both contributed to the improvement in oxidation efficiency and the reduction in CO emissions. Additionally, these results demonstrate that oxygen rich nanoparticles may contribute to improving the combustion quality and provide cleaner biodiesel-diesel engine operation^59,60^.

#### Smoke opacity

**Fig. 12:**
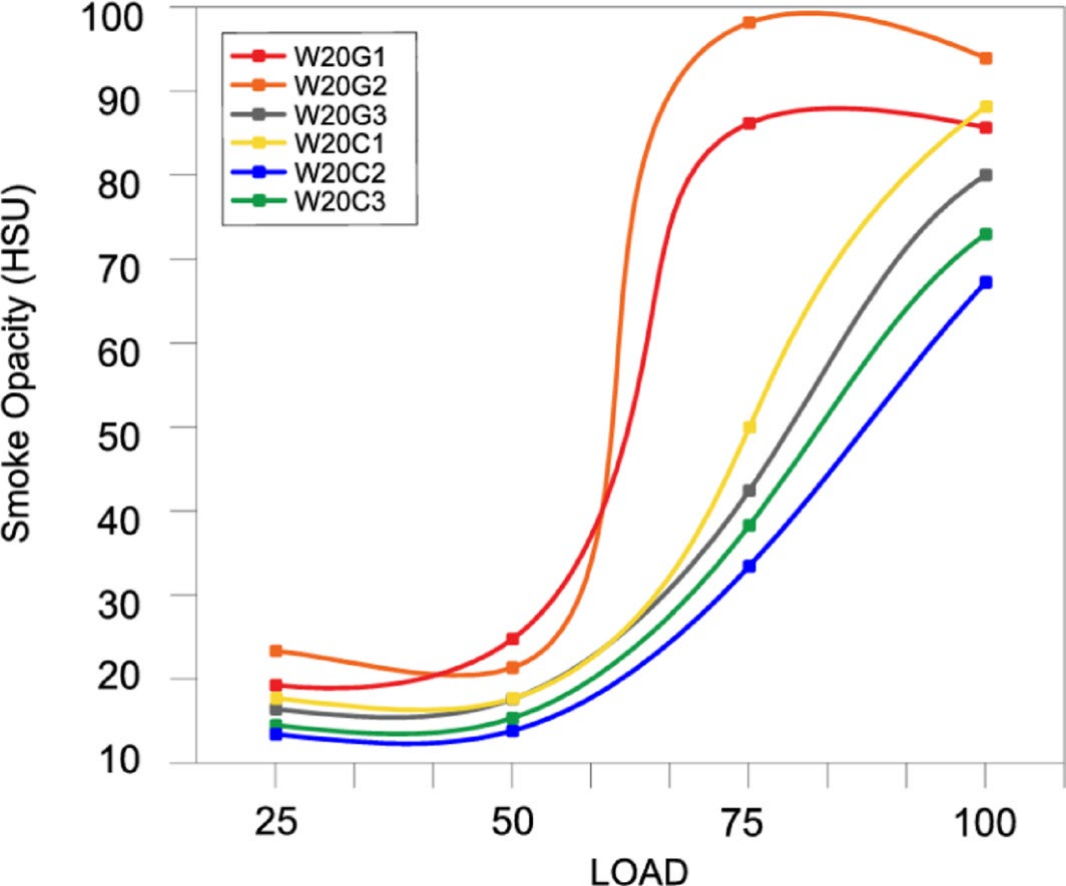
Impact of varying injection pressures on smoke opacity.

Smoke opacity emissions were reduced while operating the engine at lower loads. Smoke opacity values were measured for blends W20G1, W20C1, W20G2, W20C2, W20G3 and W20C3 at 12.6 HSU, 13.6 HSU, 11.6 HSU, 12.7 HSU, 6.9 HSU, and 8.2 HSU respectively. A comparison of the effect of different fuel blends on exhaust smoke opacity can be found in Fig. 12. Smoke opacity increases proportionally with engine load because of higher volume of fuel that is injected into the cylinder, and more difficult combustion conditions. However, increasing the fuel injection pressure results in a decrease in soot emissions for all blends. For example, the W20G1 blend was shown to have smoke opacity values approximately 58% higher when the injection pressure was lowered from 240 bar to 180 bar, illustrating the large impact that the fuel injection pressure has on the formation of soot. Of the blends that were tested, W20G3 exhibited the lowest smoke opacity values, irrespective of engine load or fuel injection pressure, indicating the excellent combustion properties imparted by the addition of graphene nanoparticles. The opposite was true for the blends containing carbon nanotubes (CNTs), where these blends exhibited the largest amounts of soot emissions. All of the blends that were tested demonstrated the highest smoke opacity value at full engine load, and this was anticipated given that there are both more fuel being supplied to the cylinder, and more difficult combustion conditions present at maximum load. The reduction in smoke opacity for the blends that contained graphene, was primarily due to the graphene’s ability to adsorb oxygen through its large surface area, promoting complete combustion, and reducing soot formation. Although CNT-containing blends did enhance the combustion performance of the fuels, they were less effective than graphene in decreasing soot emissions. These results illustrate the effectiveness of graphene nanoparticles as advantageous nano-additives to enhance combustion performance and to mitigate particulate matter emissions in biodiesel-blended fuels.

## Conclusion

This study investigated the influence of graphene and carbon nanotube (CNT) nanoparticle additives on the combustion, performance, and emission characteristics of a diesel engine operating with B20 biodiesel under varying injection pressures. Based on the experimental findings, the following conclusions can be drawn:

- Nanoparticle addition significantly improved combustion behaviour. Both graphene and CNTs enhanced fuel–air mixing, shortened ignition delay, and increased peak in-cylinder pressure and heat release rate compared to baseline B20. Graphene exhibited a marginally stronger effect due to its higher surface area and thermal conductivity.
- Engine performance showed measurable improvement at 50 ppm loading. Brake thermal efficiency increased while brake specific fuel consumption decreased for both nano-blended fuels. The optimum improvement occurred at higher injection pressures (≈ 240 bar), indicating synergistic interaction between enhanced atomization and nanoparticle-assisted combustion.
- Emissions were favourably affected, with notable reductions in CO, HC, and smoke opacity. Nanoparticles promoted more complete oxidation and soot burnout. However, a moderate increase in NOx emissions was observed, consistent with higher combustion temperatures and faster premixed burning. This trade-off may be addressed with EGR or after-treatment strategies in future work.
- Fuel stability and handling remained within acceptable limits. At the selected 50 ppm concentration, both graphene and CNT blends maintained dispersion stability during testing without causing injector fouling or measurable increases in viscosity.
- Overall, the results confirm that graphene- and CNT-enhanced B20 blends, when coupled with optimized injection pressure, can substantially improve combustion efficiency and reduce key emissions relative to conventional B20 biodiesel.

## Future directions

Future investigations should encompass a broader spectrum of nanoparticle loadings and diverse nanomaterial classes to determine optimal formulations that improve combustion performance and minimize pollutant emission. In addition, the viability of nano-additives in low-temperature combustion (LTC) engines such as HCCI (Homogeneous Charge Compression Ignition) and RCCI (Reactivity Controlled Compression Ignition) should be explored. The catalytic and ignition-enhancing properties of graphene and CNTs may help overcome the challenges of lower in-cylinder temperatures, thereby improving stability and reducing emissions under LTC operation. Finally, a thorough assessment of the potential environmental and health impacts of nanoparticles released during combustion is crucial to ensure the safe and sustainable use of these innovative fuel additives in diesel engines. To ensure the practical viability of this technology, a comprehensive investigation into the long-term engine durability and wear effects caused by nanoparticle additives and elevated injection pressures is necessary. Studying the interaction between injection pressure and advanced injection strategies, such as multiple or pilot injections, could further improve combustion control and efficiency. Additionally, integrating nanoparticle-enhanced fuels with emission control technologies like selective catalytic reduction (SCR) and diesel particulate filters (DPF) may provide synergistic reductions in nitrogen oxides and particulate matter. Detailed mechanistic studies using advanced diagnostics and modeling are also essential to better understand how nanoparticles influence combustion dynamics and pollutant formation. Finally, a thorough assessment of the potential environmental and health impacts of nanoparticles released during combustion is crucial to ensure the safe and sustainable use of these innovative fuel additives in diesel engines.

## Data Availability

The datasets used and/or analysed during the current study available from the corresponding author on reasonable request.

## Declarations

### Declaration of AI use

The authors declare that they have not use AI-assisted technologies in creating this article.
